# Emerging Biomarkers for Early Detection of Chronic Kidney Disease

**DOI:** 10.3390/jpm12040548

**Published:** 2022-03-31

**Authors:** Maja Mizdrak, Marko Kumrić, Tina Tičinović Kurir, Joško Božić

**Affiliations:** 1 Department of Nephrology and Hemodialysis, University Hospital of Split, 21000 Split, Croatia; mmizdrak@mefst.hr; 2 Department of Pathophysiology, University of Split School of Medicine, 21000 Split, Croatia; marko.kumric@mefst.hr (M.K.); tticinov@mefst.hr (T.T.K.); 3 Department of Endocrinology, Diabetes and Metabolic Disorders, University Hospital of Split, 21000 Split, Croatia

**Keywords:** chronic kidney disease, biomarker, early detection, diagnosis

## Abstract

Chronic kidney disease (CKD) is a major and serious global health problem that leads to kidney damage as well as multiple systemic diseases. Early diagnosis and treatment are two major measures to prevent further deterioration of kidney function and to delay adverse outcomes. However, the paucity of early, predictive and noninvasive biomarkers has undermined our ability to promptly detect and treat this common clinical condition which affects more than 10% of the population worldwide. Despite all limitations, kidney function is still measured by serum creatinine, cystatin C, and albuminuria, as well as estimating glomerular filtration rate using different equations. This review aims to provide comprehensive insight into diagnostic methods available for early detection of CKD. In the review, we discuss the following topics: (i) markers of glomerular injury; (ii) markers of tubulointerstitial injury; (iii) the role of omics; (iv) the role of microbiota; (v) and finally, the role of microRNA in the early detection of CKD. Despite all novel findings, none of these biomarkers have met the criteria of an ideal early marker. Since the central role in CKD progression is the proximal tubule (PT), most data from the literature have analyzed biomarkers of PT injury, such as KIM-1 (kidney injury molecule-1), NGAL (neutrophil gelatinase-associated lipocalin), and L-FABP (liver fatty acid-binding protein).

## 1. Introduction

Chronic kidney disease (CKD) is decreased kidney function defined by a glomerular filtration rate (GFR) of less than 60 mL/min/1.73 m^2^ and/or markers of kidney damage, of at least 3 months duration [1]. CKD has been recognized as a hidden epidemic and it represents a major public health problem affecting 13.4% of the adult population and causing 1.2 million deaths per year [2,3,4]. Kidney failure has a huge impact on global health as a direct cause of both morbidity and mortality globally, furthermore, as a major economic burden and also as an important risk factor for cardiovascular diseases (CVD) [5]. CVD are the most common causes of death and, in this population, end-stage renal disease (ESRD) cardiovascular mortality is estimated to be 30 times higher than in a control group [6,7]. Many of these patients are asymptomatic or have nonspecific symptoms, which makes a timely diagnosis difficult. Kidney diseases are complex and diverse and any part of the nephron can be effected, therefore, clinical assessment of kidney function largely relies on the glomerulus [8]. In clinical practice, impaired kidney function is estimated by using the glomerular filtration rate (GFR), serum levels of creatinine and cystatin C, and the presence of albuminuria. Estimations of the GFR based on the abovementioned markers are routinely used, but imprecise. There is a nonlinear correlation between creatinine/cystatin C and thee GFR and, consequently, relatively small initial increases in these markers represent significant decreases in the GFR [9]. Serum creatinine concentration increases only when approximately 40–50% of the kidney parenchyma is damaged [9,10,11]. Meanwhile, albuminuria precedes a decrease in GFR but can be absent in tubulointerstitial or hypertensive kidney diseases [12]. Furthermore, 30% of patients with diabetic kidney disease have normal urine albumin levels [13]. Because of the known limitations of these markers, several alternative markers have been studied, namely beta-trace protein (BTP) and β-2-microglobulin (B2M) [14]. Therefore, the early stages of CKD can remain underdiagnosed. It is important to highlight that treatment of CKD in the early stages can improve kidney function or at least slow down the progression of CKD. Blood and urinary markers of kidney function are increasingly being used to diagnose CKD in the early stage and consequently give appropriate therapy and improve patient management and prognosis [15]. This review article aims to summarize achievements to date about early markers of chronic kidney disease and to analyze their practical utility.

## 2. Etiopathological Aspects of Chronic Kidney Disease

**Figure 1:**
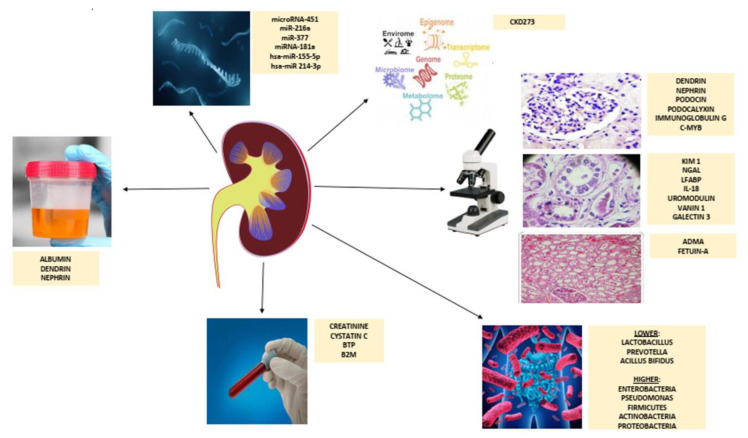
Schematic overview of diagnostic tools in an early detection of chronic kidney disease. Abbreviations: BTP, beta-trace protein; B2M, beta-2-microglobulin; KIM-1, kidney injury molecule-1; NGAL, neutrophil gelatinase-associated lipocalin; LFABP, liver fatty acid-binding protein; IL-18, interleukin 18; ADMA, asymmetric dimethylarginine.

Diabetic nephropathy and nephroangiosclerosis due to arterial hypertension are the most common causes of chronic kidney disease. The rapid rise of common risk factors such as diabetes, hypertension, obesity, and glomerular diseases in high-income countries, as well as infections especially among the poor, will result in even greater and more profound burdens associated with CKD. A kidney biopsy is a golden standard in the diagnostic algorithm of kidney disease because it determines a pathohistological diagnosis and also a proportion of acute and chronic changes relevant to the prognosis [16]. The percentage of segmental and global glomerulosclerosis, interstitial fibrosis, and tubular atrophy determine the chronicity. In a kidney biopsy that is performed early, these histological parameters may be absent, and the long-term prognosis of renal function remains unclear. Therefore, it would be useful to recognize noninvasive biomarkers that are included in the very early activation of the fibrotic process to therapeutically prevent remodeling of renal tissue. Irrespective of the etiology of CKD, various structural and functional changes within the kidney that develop during the disease course can result in glomerular, tubulointerstitial, and vascular injuries [14]. In diseases with predominant glomerular affection, due to the downstream position of tubules and because of changes in blood flow and the quality/quantity of ultrafiltration, secondary tubulointerstitial damage occurs [17]. The advanced stage of the disease is characterized by a persistent state of inflammation, hypoxia, and oxidative stress that contribute to the development of renal fibrosis which is a unique irreversible pattern of all chronic kidney disease [14]. Table 1 summarizes different biomarkers of impaired kidney function regardless of their roles which might act as early markers of renal deterioration. In the further text, due to their abundance, only several of the most promising are analyzed in more detail (Figure 1).

## 3. Biomarkers of Glomerular Injury

### 3.1. Dendrin

Dendrin is a proline-rich protein originally identified in telencephalic dendrites of sleep-deprived rats [20]. In addition to brain dendrin, dendrin is only found in the kidneys, linearly expressed in podocytes along with glomerular capillary loops [21]. It is an integral part of the slit diaphragm complex, and it contributes to regulation of podocyte function and is involved in the glomerular filtration process [22]. In response to glomerular injury and upregulated TGF-β, dendrin relocates from the membrane to the nucleus, thereby, promoting apoptosis [22,23,24]. Nuclear dendrin acts as a transcriptional factor of cytosolic enzyme cathepsin L, which proteolyzes CD2-associated protein, thereby, increasing the apoptotic susceptibility to pro-apoptotic TGF-β [25]. Due to this fact, nuclear relocation of dendrin in glomerular diseases might be a marker of disease [22,23].

The expression of dendrin in podocyte nuclei has been reported by studies on different glomerulopathies: focal segmental glomerulosclerosis, lupus nephritis, membranous nephropathy, IgA nephropathy (IgAN)/IgA vasculitis, and minimal change disease [22,23,24,25]. Dendrin expression might be irreversibly switched off in chronically damaged glomeruli [24]. In our study, a higher proportion of dendrin negative glomeruli was significantly correlated to lower CC both at the time of biopsy and follow-up [24]. Kodama et al. reported that there were an increasing number of dendrin-positive nuclei in the glomeruli suggesting acute glomerular injury, as well as apoptotic podocytes were detectable in the urine of IgAN patients [25].

### 3.2. Nephrin

Nephrin is a 180 kD transmembrane protein expressed in glomerular podocytes where it has a pivotal role in glomerular filtration barrier formation and in maintaining its function [26]. It is expressed on the lateral aspect of podocyte foot processes [27]. The main role of nephrin is to prevent the passage of protein through the glomerular barrier regardless of disease type. It was first identified in children with congenital nephrotic syndrome of the Finnish type [28]. Early events in the damaged podocytes are alterations of the slit diaphragm, reorganization of the foot process structure with fusion of filtration slits and apical displacement, and finally, detachment from the glomerular basement membrane [29]. These changes can lead to severe and progressive glomerular injuries present in different glomerular diseases: minimal change disease, membranous glomerulopathy, crescentic glomerulonephritis, collapsing glomerulopathy, focal segmental glomerulosclerosis, diabetic nephropathy, and lupus nephritis [26]. All these podocytopathies result in the detection of nephrin in the urine [26]. According to data from the literature, urinary nephrin is a more sensitive biomarker than albuminuria in the early detection of diabetic nephropathy [30]. Hyperglycemia alters nephrin expression, its phosphorylation, and finally downregulation, as well as causes podocyte disruption. Nephrinuria is present in 100% of diabetic patients with micro and macroalbuminuria and about 50% of normoalbuminuric patients, which demonstrates that it may precede microalbuminuria. Furthermore, the urinary nephrin proportionately increases from normoalbuminuria to macroalbuminuria. This might emphasize the role of podocyte metabolism in diabetic kidney disease [31,32,33].

### 3.3. Podocin

Podocin is a 43 kDa membrane-associated protein, which is a crucial component of the glomerular slit diaphragm. It plays an important role in nephrin-mediated cellular signaling and assures podocyte structure and function [34]. Podocin mutations cause a spectrum of kidney disorders, ranging from neonatal nephrotic syndrome to late-onset focal segmental glomerulosclerosis. Experimental studies on mice have shown that the absence of podocin led to a rapidly progressive renal disease characterized by mesangiosclerosis, glomerulosclerosis, tubulointerstitial damage, and nephrotic syndrome [35].

Mollet et al. discovered that podocin knockout mice had a gradient of glomerular lesions that demonstrated a developmental stage dependence of renal histologic patterns of injury [35]. Significant albuminuria occurred only after early and focal foot process effacement had progressed to diffuse involvement, with the complete absence of podocin immunolabeling at the slit diaphragm [35]. According to data from the literature, on the one hand, podocin might be an early biomarker of diabetic nephropathy associated with the severity of the disease [36,37]. The results have shown that podocin was higher in patients with diabetes mellitus as compared with a healthy group regardless of the level of albumin to creatinine ratio, even in those with normoalbuminuria. On the other hand, GFR and serum albumin showed negative correlations with urinary podocin and a positive correlation with serum creatinine [36,37]. Furthermore, detection of podocytes in the urinary sediments might be a marker of severe injury and disease activity [38]. A comparison of the presence of urinary podocytes that they were absent in healthy controls, diabetic patients with normoalbuminuria, and diabetic patients with chronic renal failure; however, they were detectable in those with microalbuminuria and macroalbuminuria [38].

### 3.4. Podocalyxin

Podocalyxin (PCX) is an anionic transmembrane sialoglycoprotein, which is a member of the CD34 protein family [39]. It is expressed on the apical side of podocyte foot processes and is an important component of the slit diaphragm structure [27]. Therefore, urinary podocalyxin might act as a biomarker of podocyte dysfunction that might display the integrity of the kidney’s filtration barrier [27]. It has been analyzed in different types of kidney diseases. Although the diagnostic potential of urinary podocalyxin for chronic kidney disease is still not completely understood, the preliminary data are promising. The results have shown that urine podocalyxin was elevated in patients with diabetes mellitus, therefore, being a more sensitive and specific biomarker in the early detection of diabetic nephropathy than albuminuria [39].

Furthermore, in other glomerular diseases, the level of urinary podocalyxin and the number of urinary podocytes were associated with the proportion of segmental sclerosis [40]. Expression of urinary PCX mRNA correlated with the standard biomarkers of kidney function assessment (serum creatinine, eGFR, and albuminuria) [41,42]. It was also significantly increased in obese children making it a potential sensitive marker of obesity-related kidney disease in children [43]. In patients with systemic erythematosus lupus, the podocalyxin creatinine ratio was higher in patients with lupus nephritis than in those without the disease [44]. It also correlated with histological features of the disease, being higher in the proliferative form of the disease [44].

### 3.5. Immunoglobulin G

Immunoglobulin G (IgG) antibody is a 150 kDa globular protein, which is a major component of humoral immunity. There are structural differences among four human IgG subclasses meritorious for different biologic effector functions. In kidney disease, due to its size, when the selectivity of the glomerular capillary wall is severely disrupted, IgG will be filtered from the blood and excreted in the urine [45]. Because of that fact, the IgG can be a marker for mechanical injury of the glomerular filtration barrier [45]. According to data from the literature, higher urine and lower serum IgG levels have been associated with a higher proportion of chronic pathological changes, lower estimated GFR, and poor renal outcome [45,46]. In the immunohistochemical study of IgA nephropathy, co-dominant IgG with IgA deposition was a weaker indicator that correlated with dysregulated arterial hypertension and higher proteinuria [47]. On the contrary, the IgG deposits in necrotizing crescentic glomerulonephritis associated with ANCA antibodies were not related to adverse renal clinical outcomes [48]. However, most of the studies that have been conducted analyzed diabetic nephropathy. Urinary excretion of IgG was significantly increased in diabetic patients including even those with normoalbuminuria as compared with healthy controls [49,50,51]. Hence, it might be a more sensitive biomarker than albuminuria for detecting the early stages of diabetic nephropathy [49,50,51]. Finally, since IgG glycosylation is an important post-translation process with a pathophysiological role in diabetic nephropathy progression, IgG N-glycosylation patterns have been associated with a faster decline of kidney function. Estimated GFR, but not albumin to creatinine ratio, is associated with IgG glycans, which suggests these correlations may represent renal macroangiopathy rather than a microvascular disease [52].

### 3.6. c-Myb

c-Myb is a DNA-binding transcription factor. Postnatally, it is included in the normal control of differentiation and regulation of hematopoietic and epithelial cells in many organs, including the kidneys, but mutated c-Myb has a role in tumorigenesis [53]. c-Myb regulates Slug protein and products of SNAI1 and SNAI2 (Slug) directly repress transcription of E-cadherin and other junctional proteins, triggering desmosome disruption and cell spreading which contributes to epithelial–mesenchymal transition and consequently fibrosis [54,55,56]. The reactivation of Snail and Slug in some renal diseases is associated with fibrosis progression [56]. In our study, c-Myb was, for the first time, analyzed immunohistochemically in kidneys of patients with IgAN/IgA vasculitis and healthy controls. c-Myb was expressed in glomerular epithelial cells, as well as distal tubules [57]. Patients significantly differed from controls, in that c-Myb expression was higher through the entire nephron of patients. However, regardless of creatinine clearance at the time of biopsy, patients differed from the control group in c-Myb expression, highlighting c-Myb as a potential novel biomarker of early kidney damage. Further studies on a larger cohort of different glomerular diseases are needed to confirm these results.

## 4. Biomarkers of Tubulointerstitial Injury

### 4.1. Kidney Injury Molecule-1

Kidney injury molecule-1 (KIM-1) is a 38.7 kDa type I transmembrane glycoprotein, with an extracellular immunoglobulin-like domain [58]. KIM-1 also has a role as a phosphatidylserine receptor that transforms epithelial cells into semi-professional phagocytes [59]. It is expressed at low levels in the kidney and other organs; however, it is significantly upregulated in kidney injury, especially after an ischemia-reperfusion injury, in some renal tubulointerstitial diseases, and polycystic kidney disease [58,60]. A soluble form of human KIM-1 has been detected in the urine of patients with acute tubular necrosis shortly after injury, which correlated with the degree of the injury [60,61]. Because of these facts, KIM-1 may act as a biomarker for renal proximal tubule injury and the associated recovery processes [58,60,61].

In the context of chronic kidney disease, KIM-1 is also a sensitive biomarker for chronic proximal tubular injury, which is correlated with the incidence, progression, and prognosis of CKD [60]. Continued chronic expression of KIM-1 in renal tubules promotes the secretion of monocyte chemotactic protein 1 and consequentially stimulates proinflammatory milieu and fibrosis [60]. In a study on the most common type of CKD, i.e., diabetic nephropathy, in early stages, the expression of KIM-1 in the glomeruli was significantly elevated, mainly in the proliferative parietal epithelium of the capsule [60]. Its expression increased along with the development of the disease and correlated with podocytopenia and proteinuria [60].

### 4.2. Neutrophil Gelatinase-Associated Lipocalin (NGAL)

Neutrophil gelatinase-associated lipocalin (NGAL) (also siderocalin, lipocalin-2 (LCN2), or lipocalin) is a ubiquitous 21–25 kD iron-carrying protein of the lipocalin superfamily, highly expressed in the tubular epithelium of the loop of Henle and collecting ducts [62]. Initially, it was found in activated neutrophils, and under physiological conditions, it has a role as an innate antibacterial factor [63]. NGAL is one of the first molecules to trigger kidney development, converting embryonic mesenchymal cells into epithelial cells forming tubules and complete nephrons [64].

Early release of NGAL from tubular epithelial cells occurs following damage [62,65]. The level of NGAL expression correlates with the degree of kidney injury and may help to discriminate patients who are at higher risk of faster decline in kidney function [63,64,66]. Expression of NGAL results in enhanced cell proliferation, cytogenesis, renal damage, and CKD progression [64]. NGAL has shown a good correlation with estimated GFR, cystatin C, and serum creatinine [62]. Further, urinary NGAL is a good predictor of renal injury before detectable changes in eGFR, as well as a marker of normoalbuminuric renal disease in type 2 diabetes mellitus [64,67].

### 4.3. Liver Fatty Acid-Binding Protein (L-FABP)

Liver fatty acid-binding protein (FABP), also known as L-FABP or FABP1 is a 14 kDa soluble protein found predominately in the cytoplasm of hepatocytes, enterocytes, renal proximal tubular cells, and alveolar epithelium [68]. Interestingly, FABP1 is present in humans, but not mouse kidneys [68]. Under physiological conditions, albumin is filtered from the glomeruli and reabsorbed predominantly in the proximal tubules bound to free fatty acids. After reabsorption, cytosolic albumin releases fatty acids to L-FABP and moves into lysosomes during this process [69]. L-FABP binds long-chain fatty acids, which plays a role in the fatty acid metabolism, intracellular signaling, and promotes the excretion of lipid peroxidation products, achieving renoprotection [69].

In CKD patients, fatty acids overload the proximal tubule, and massive proteinuria is found [69]. L-FABP expression and urinary excretion are increased by various stressors, such as proteinuria, hyperglycemia, tubular ischemia, toxins, and salt-sensitive hypertension [70]. Urinary L-FABP levels accurately reflect the degree of tubulointerstitial damage and are significantly correlated with the prognosis and progression of CKD [70]. Moreover, according to data from the literature, L-FABP showed a lower interference by leukocyturia and hematuria than NGAL [71]. In diabetic patients, regardless of type, urinary L-FABP levels are higher in patients with normoalbuminuria than in those with microalbuminuria, thus, reflecting early stages of diabetic nephropathy [70].

### 4.4. Interleukin 18

Interleukin (IL)-18, also known as the interferon-gamma inducing factor, is a member of the IL-1 superfamily. It is a proinflammatory cytokine that strongly induces a Th1 response. IL-18 can modulate both innate and adaptive immunity and its dysregulation can cause autoimmune or inflammatory diseases. IL-18 is stored intracellularly as a biologically inactive 24 kDa precursor and is secreted extracellularly as an 18 kDa bioactive mature molecule after being cleaved by caspase-1 [72]. Different types of cells produce IL-18, in renal tubular epithelial cells. IL-18 is a biomarker that has the ability to differentiate acute tubular necrosis from other etiological factors of renal disease [73,74]. In chronic kidney disease, IL-18 is overproduced and might be associated with the promotion and progression of fibrosis [75].

According to data from the literature, an elevated IL-18 level was associated with diabetic nephropathy, and has been reported to be a predictive marker for the development of disease, degree of albuminuria, and decline of kidney function [76,77,78]. In renal biopsies from ANCA-associated vasculitis patients, IL-18 positivity was found in podocytes and fibroblasts, distal tubular epithelial cells, and interstitial macrophages [79]. In patients with IgA nephropathy, serum IL-18 levels correlated significantly with proteinuria, the estimated glomerular filtration rate, chronic IFTA, and renal function during follow-up [80].

### 4.5. Uromodulin

Uromodulin or Tamm–Horsfall protein is a glycoprotein expressed only by renal tubular cells of the thick ascending limb of the loop of Henle and distal tubule [81]. It is physiologically present in urine in large aggregates where it has several roles: constitutive inhibitor of calcium crystallization; defender against urinary tract infections via its mannose-containing side chains, renal ion transporter, and immunomodulator; and finally, possibly as a systemic antioxidant [82]. Opposite to the conventional view of uromodulin as an instigator in kidney injury, El-Achkar et al. investigated uromodulin knockout mice and proposed an important role of uromodulin in protection from kidney injury by downregulating inflammation [83]. On the contrary, Lhotta et al. reported that lower genetically determined urinary uromodulin concentrations protected against renal disease and CKD was associated with higher serum levels of uromodulin [81]. It is hypothesized that uromodulin entering the renal interstitium, either by basolateral secretion or urinary back-leakage in damaged tubuli, interacts with and stimulates cells of the immune system and, thereby, causes inflammation and progression of chronic kidney disease [81].

Uromodulin activates specific components of the immune system, and thus, may act as a signaling molecule for renal tubular damage [84]. Estimated glomerular filtration correlated with urinary uromodulin and negatively correlated investigating uromodulin knockout mice with serum uromodulin. Patients with both very low urinary and serum uromodulin had the highest tubular atrophy scores [84]. Recent studies on uromodulin have shown that its concentrations in CKD patients were lower than in healthy subjects, and the lower concentrations were associated with more advanced stages of CKD [85]. Uromodulin levels were positively associated with estimated GFR and inversely associated with proteinuria, as well as independently associated with ESRD or rapid loss of estimated GFR [86].

### 4.6. Vanin 1

Vascular non-inflammatory molecule-1 (vanin 1) is a glycosylphosphatidylinositol-anchored ectoenzyme with pantetheinase activity [65,87]. It has different roles such as the recycling of pantothenic acid (vitamin B5), which is an important precursor in the biosynthesis of coenzyme A, in energy production, but also in oxidative stress and inflammation [87]. It is highly expressed in the liver, intestine, and kidneys [87]. Experimental studies on rats have detected urinary vanin 1 in an early stage of hypertensive CKD, as well as an early biomarker for renal tubular damage in normotensive rats under a high-salt intake [65,88]. Vanin 1 also plays a role in kidney damage in a rat model of type 1 diabetic nephropathy [87]. Renal expression of vanin 1, at both the gene and protein levels, is modulated differently depending on the specific etiology of the injury and potential correlation with diseases [87]. In human studies, urinary vanin 1 was an early biomarker of kidney injury associated with CKD and an independent risk factor of kidney function decline in hypertensive patients [65,89].

### 4.7. Galectin-3

Galectin-3 is a 32–35 kDa member of the galectin family of β-galactoside-binding lectins, which is characterized by a carbohydrate recognition domain [90]. It is detected in endothelial cells, epithelial cells, and macrophages. It has an important function in numerous biological activities including cell growth, apoptosis, pre-mRNA splicing, differentiation, transformation, angiogenesis, adhesion, immunity, inflammation, and fibrosis [91]. An increase in the concentration of galectin-3 is associated with kidney fibrosis, increased risks of rapid renal function decline, incident chronic kidney disease, and progressive renal impairment, as well as cardiovascular endpoints, infection, and all-cause mortality in patients with renal failure [92]. Therefore, galectin-3 not only protects renal tubules from chronic injury by limiting apoptosis but that may lead to enhanced matrix remodeling and fibrosis attenuation [93]. The results of the recent investigation have shown that galectin-3 levels were significantly elevated in type 2 diabetes mellitus patients with macroalbuminuria, and higher levels of galectin-3 were found in patients with advanced kidney function (stage 4 and 5 CKD) [94].

## 5. The Role of Omics in Early Detection of CKD

Respecting the fact that standard laboratory markers of deteriorated kidney function are scarcely affected in early stages, several targeted strategies have been designed to search for novel early specific biomarkers of CKD diagnosis. Although not routinely, omics techniques are slowly being recognized for their roles in early CKD diagnosis. For instance, proteomics has already been found to be valuable for identifying anti-M-type phospholipase A2 receptor autoantibody in the diagnosis of primary membranous nephropathy (as many as 70% of cases) [95]. Omics technologies permit high-throughput, comprehensive exploration of the genome, epigenome, proteome, transcriptome, and metabolome [96]. The goal of metabolomics is to identify nontargeted, global small-molecule metabolite profiles of complex samples [97]. Chen et al. analyzed metabolomics in 2155 patients with stages 1–5 CKD and healthy controls and identified five metabolites, whose levels were associated with kidney disease [92], i.e., 5-methoxytryptophan (5-MTP), canavaninosuccinate (CSA), acetylcarnitine, tiglylcarnitine, and taurine [92]. For example, 5-MTP levels decreased with progression of CKD [92]. Overexpression of tryptophan hydroxylase-1 (TPH-1), an enzyme involved in 5-MTP synthesis, reduced renal injury by attenuating renal inflammation and fibrosis, whereas TPH-1 deficiency exacerbated renal injury and fibrosis by activating NF-κB and inhibiting Nrf2 pathways [92]. Furthermore, biomarkers associated with early detection of CKD are single nucleotide polymorphisms in the MYH9/APOL1 and UMOD genes, the proteomic CKD273 biomarker panel, and metabolite pantothenic acid [98]. Capillary electrophoresis coupled with mass spectrometry has been used to develop a proteome-based urine biomarker panel of 273 urinary peptides with profiles that differed significantly between individuals with CKD and healthy controls (CKD273 risk score) [Good, 2010]. CKD273 is commercially available as an in vitro diagnostic test for early detection of CKD [99]. The multicenter, prospective observational study with embedded randomized controlled trial (PRIORITY) from 15 specialist centers in ten European countries tested whether CKD273 was associated with development of microalbuminuria and whether progression to microalbuminuria could be prevented with the mineralocorticoid receptor antagonist spironolactone [100]. The results showed that in people with type 2 diabetes and normoalbuminuria a high-risk score from CKD273 was associated with an increased risk of progression to microalbuminuria over a median of 2.5 years, independently of clinical characteristics [100].

In an extensive study by Romanova et al., the results showed that blood levels of IL-1β, IL-2, IL-4, IL-5, IL-6, IL-7, IL-8, IL-9, IL-10, IL-12 (p70), IL-13, IL-15, IL-17, Eotaxin, FGFb, G-CSF, GM-CSF, IP-10, MCP-1, MIP-1α, MIP-1β, PDGF-1bb, RANTES, TNF-α, and VEGF were significantly higher in patients with CKD as compared with a control group, and they were positively correlated with kidney function [101]. The multiple reaction monitoring quantification method revealed that serum levels of HSP90B2, AAT, IGSF22, CUL5, PKCE, APOA4, APOE, APOA1, CCDC171, CCDC43, VIL1, Antigen KI-67, NKRF, APPBP2, CAPRI, and most complement system proteins were also elevated in CKD patients as compared with a healthy control group [101]. The authors, however, highlighted that only AAT and HSP90B2 correlated with standard markers of kidney function and might be CKD biomarkers [101].

Nevertheless, implementation of omics in the contemporary diagnostic pathway of early CKD diagnosis also bears some notable limitations. Firstly, since a sample collected for proteomics is just a snapshot of an active ongoing process, repeated sample collection might be required to ensure consistency. Furthermore, as proteomics methods are multilayered, appropriate analytical approaches and external validation in separate cohorts are paramount to verify findings from omics studies before implementation to clinical practice. Finally, perhaps the most important consideration for translation of proteomics to clinical use is high cost. In this sense, it is valuable to address that health economic analyses have indicated the cost-effectiveness of CKD273, however, no strategies exist to cover the costs for widespread applications of this method.

## 6. Microbiota and CKD

Human intestines contain over 1014 microorganisms that can be classified as beneficial, harmful, or neutral, and are more than 10 times the total number of human cells [102]. Intestinal flora play important roles in host metabolism, digestion, immunity, and barrier protection, as well as the pathophysiology of a spectrum of diseases including CKD [102]. CKD progression is associated with alterations in gut microbiota, and therefore, changes in gut microbiota may be helpful in the early detection of CKD. According to data from the literature, the number of probiotic bacteria (*Lactobacillus*, *Prevotella*, and *Bacillus bifidus*) is lower in CKD patients, while the numbers of optionally pathogenic bacteria (*Enterobacteria* and *Pseudomonas*) or *Firmicutes*, *Actinobacteria*, and *Proteobacteria* are increased [102,103]. For example, *Ruminococcus* and *Roseburia* display the highest diagnostic values for distinguishing an early-stage CKD patient from healthy controls [104]. Furthermore, there is low gut microbiota diversity in patients with CKD.

Maladaptation of microflora to intestinal environmental changes can be detected at an early stage of CKD [103]. Such gut dysbiosis, followed by impaired intestinal barrier function cause the proinflammatory milieu in CKD by accommodating bacterial translocation and the presence of endotoxin and other noxious luminal products in the circulation [103,105]. Furthermore, intestinal microbiota also have a role in the endocrine system by producing diet-derived bioactive metabolites [103]. Wu et al. proposed several microbial genera, including *Escherichia-Shigella*, *Parabacteroides*, *Roseburia*, rectale_group, *Ruminococcaceae_NK4A214_group*, *Prevotellaceae_UCG-001*, *Hungatella*, *Intestinimonas*, and *Pyramidobacter*, as distinguishers between CKD and a control group [104]. Further examination has also revealed that fatty acid and inositol phosphate metabolism were enriched in CKD patients, while aminoacyl-tRNA biosynthesis; oxidative phosphorylation, phenylalanine, tyrosine, and tryptophan biosynthesis; thiamine metabolism; pantothenate, and coenzyme A biosynthesis; as well as valine, leucine, and isoleucine biosynthesis were enriched in a healthy group [104]. The intestinal microflora ferment undigested products that reach the colon, therefore, producing indoles, phenols, and amines that are further absorbed by the host and act as real uremic toxins. They have an important role in inflammation and oxidative stress generation and the pathogenesis of CKD, complications cardiovascular disease, anemia, or mineral bone metabolism disorders [105]. The most recent findings have highlighted the novel use of the circulating microbiome profile. Authors of a pilot study concluded that *Proteobacteria phylum*, *Gammaproteobacteria* class, as well as *Enterobacteriaceae* and *Pseudomonadaceae* families were more abundant in the CKD population as compared with the control group, and GFR correlated negatively with the amount of *Proteobacteria* [106].

## 7. MicroRNA in Early Detection of CKD

MicroRNA (miRNA) is a short single-stranded non-coding RNA molecule involved in RNA silencing and post-transcriptional regulation of gene expression [107]. MicroRNAs have great potential to be sensitive and specific biomarkers enabling a ”personalized medicine” approach in kidney disease as they are tissue-specific and stable in various biological materials [108]. Preliminary data have shown that microRNA-451 was an early predictor of CKD in diabetic nephropathy [109]. Estimated GFR showed a positive correlation with urinary microRNA-451 and a negative correlation with both plasma microRNA-451 and urinary albumin [109]. Previous studies have shown that an increase in uE miR-451 predicted albuminuria in diabetic rats [110].

Urinary miR-216a has also been reported to be significantly lower in all patients with type 1 diabetes, with the lowest levels among the microalbuminuria group. Further, positive correlations have been found between urinary miR-377 and albumin to creatinine ratio, while urinary miR-216a was negatively correlated to this variable [111]. According to Khurana, miRNA-181a might be the most robust and stable biomarker, being significantly decreased by about 200-fold in CKD patients as compared with in healthy controls [112].

In a subpopulation of chronic glomerulonephritis (CGN) patients, there was a higher level of expression in the urine of hsa-miR-155-5p, hsa-miR 214-3p, hsa-miR-93-5p, and hsa-miR-196a-5p in CGN with daily protein excess <3.5 g. Increased levels of expression of hsa-miR-155-5p, hsa-miR-214-3p, hsa-miR-200a-5p, and hsa-miR-29-5p have been found in CGN patients with eGFR > 60 mL/min [113]. Although their use in routine clinical practice is still not applied, miR-103a-3p, miR-192-5p, the miR-29 family, and miR-21-5p have, according to data from the literature, the greatest potential to result in novel therapeutic and diagnostic strategies [114].

## 8. Challenges in Early CKD Diagnosis

At first, it seems obvious that finding a specific biomarker that reflects an early stage of CKD and confirming a timely diagnosis would enable improved treatment and reduce the medical and financial burdens associated with CKD. However, multiple experts agree that the problem is much deeper and that pursuing that goal may be a double-edged sword, such as in population screening employed for colorectal cancer screening. In theory, CKD detection at the asymptomatic phase would result in prevention of kidney failure. However, detection of CKD may not lead to changes in management of the patient. Tonelli et al. even provided us with factors that would diminish the putative benefits of implementing an early CKD detection strategy [115]. Nevertheless, it is worth mentioning that certain populations may benefit from these strategies, such as populations with a high prevalence of underlying causes that may benefit from specific strategies (e.g., glomerulonephritis in the Japanese population) [116]. The disadvantages of early detection are also often neglected. In addition to the adverse outcomes related to unnecessary invasive procedures, CKD early diagnosis is burdened by several other potential adverse outcomes. For instance, imaging which was indicated owing to early diagnosis may detect abnormalities of questionable clinical significance (e.g., solitary cysts or incidentalomas) which then trigger further investigation and/or treatment. Again, this could seem beneficial, however, examples from real-life practice imply differently. Furthermore, multiple specialist consultations, follow-up visits, and testing lead to increased expenses, patient discomfort, and anxiety [117,118]. Moreover, if medications are prescribed, they also bear a low, but not negligible risk of severe adverse outcomes. Finally, “labeling” a patient with chronic CKD may have lifelong implications for medical insurance, work performance, and choice of occupation [119,120]. In summary, early CKD diagnosis methods should be pursued, however, we must be very cautious in implementing these methods (Whom to test? When to test?). In this sense, it may be prudent to integrate new methods with existing management programs for diseases that usually coexist with CKD (diabetes, hypertension).

Despite the considerable progress that has been made and the limitations that standardized kidney function estimation offers, currently, the biomarkers of kidney function that are most often used are creatinine and microalbuminuria. Why is it so? From the bench to the bedside, biomarker translation is rather challenging and a tedious process. Nevertheless, perhaps, the biggest setback of CKD biomarker evaluation is the lack of a reliable comparator (i.e., the golden standard). Most clinicians argue that a kidney biopsy is, for the most part, suitable, however, they are well aware of the associated risks of this invasive procedure, and also the “trap” of a diagnosis that does not affect management. Hence, comparing biomarker serum/urine levels with kidney changes assessed by biopsy is relatively rarely performed and, most commonly, an analysis is performed with creatinine (in the form of eGFR) and microalbuminuria as reference points. This is very problematic for the establishment of reliable biomarkers, simply because neither eGFR nor microalbuminuria are reliable indicators of kidney function, especially at the early phase of CKD. For instance, we previously mentioned that 30% of patients with diabetic kidney disease have normal urine albumin levels [13]. In addition, the margin of error for basically all eGFR (regardless of whether creatinine or cystatine is used) is unacceptably high across the whole spectrum of kidney function, from normal renal function to advanced CKD [121]. The discrepancy between eGFR and measured GFR is especially present in early stages of CKD, and when assessing decline in GFR. Hence, if a putative biomarker correlates with eGFR, what can we really conclude about a patient’s kidney status? Therefore, before considering a biomarker for implementation in practice, a comparison with a reliable GFR indicator is mandatory. In this sense, Luis-Lima et al. recently simplified the measurement of plasma clearance of iohexol, a reliable GFR indicator, by replacing venous blood samples with dried capillary blood samples deposited on filter paper, thus, creating a simpler but precise method of GFR estimation [122].

## 9. Conclusions

In this review article, several biomarkers were listed. The review of the relevant literature identified hundreds of biomarkers, some of which have been thoroughly examined, however, there is still no biomarker that has been incorporated into routine clinical practice as a unique biomarker. Owing to multiple aforementioned limitations, it is probable that this goal will not be achieved in the near future. Although an omics analysis provides a novel approach to personalized medicine and has promising results in CKD, it also bears substantial limitations, especially with respect to high costs. From a clinical standpoint, perhaps the first step should be to determine the population of individuals at high-risk for developing CKD based on integrative and critical assessments of patients, whereas from a preclinical standpoint, the first goal is to use an appropriate comparator (i.e., the golden standard) for assessing the use of putative biomarkers in the early diagnosis of CKD.

## Figures and Tables

**Table 1:** Summary of possible biomarkers of chronic kidney disease according to their roles [14,18,19].

Glomerular	Tubulointerstitial	Cardiovascular	Endothelial Function
Albumin	Cystatin C	Natriuretic peptids	ADMA
Immunoglobulin G	KIM-1	Cardiac troponin T	Fetuin A
Dendrin	NGAL	C-reactive protein	Uric acid
Nephrin	NAG	Adiponectin	
Transferrin	H-FABP	Lectin	Inflammation
Type IV collagen	CTGF	FGF 23	Interleukin 6
Fibronectin	c-Myb	Klotho	Pentraxin 3
Laminin	Uromodulin	Calciprotein particle	TNF-α
Podoplanin	IL-18	Wingless (Wnt) antagonists Inhibitors	Interleukin-1β
Sinaptopodin	Galectin 3	PGDF-15	IP-10
Glycosaminoglycans	Vanin 1	Paraoxonase 1	MCP-1
Ceruloplasmin	Nestin	Adrenomedullin	CD 14 mononuclear cells
L-PGDS	α1-Microglobululin		Tenascin
Immunoglobulin M	TIMP-1	Oxidative stress	Interleukin 8
Desmin		8oHdG	YKL-40
SMAD 1		AGEs	sCD40L
Podocalyxin		Pentosidine	CHIT1
ADAM 10			YKL-40
Glepp-1			
α-Actinin 1			RAAS activation
VEGF			Angiotensionogen
c-Myb			
Podocin			
β-Enolase			

L-PGDS, lipocalin-type prostaglandin D synthase, ADAM 10, ADAM metallopeptidase domain 10; VEGF, vascular endothelial growth factor; KIM-1: kidney injury molecule-1; NGAL, neutrophil gelatinase-associated lipocalin; NAG, N-acetyl-beta-D-glucosaminidase; H-FABP, heart-type fatty acid binding protein; CTGF, connective tissue growth factor; IL-18, interleukin 18; TIMP-1, tissue inhibitors of metalloproteinases-1; FGF 23, fibroblast growth factor 23; PGDF-15, plasma growth differentiation factor-15; 8oHdG, 8-oxo-2′-deoxyguanosine; AGEs, advanced glycation end products; ADMA, asymmetric dimethylarginine; TNF-α, tumor necrosis factor α; IP-10, inducible protein 10; MCP-1, monocyte chemoattractant protein-1; YKL-40, chitinase 3-like 1; CHIT1, chitotriosidase-1.

## Data Availability

Not applicable.
